# Lectures on Diabetes: Including the Bradshawe Lecture Delivered before the Royal College of Physicians on August 18th, 1890

**Published:** 1891-06

**Authors:** 


*Lectures on Diabetes: including the Bradshawe Lecture delivered before the Royal College of Physicians on August* 18*th*, 1890. By ROBERT SAUNDBY, M.D. Edin., F.R.C.P. Lond. Bristol: JOHN WRIGHT & CO. 1891.

In this book, which is distinguished by the clearness of statement and thoroughness of treatment which marked the *Lectures on Bright's Disease* by the same author, we have an account of the etiology, morbid anatomy, symptomatology, and treatment of diabetes which fully comes up to the anticipations we had formed from our knowledge of his previous work, and which indeed leaves little or nothing to be desired. In it the latest researches and the older established facts about the disease will be found stated in due order, stress being properly laid on the most important points, whilst the minor ones are not neglected, and the whole subject treated in such a way that the orderly arrangement impresses the chief features of diabetes upon the memory. The author has the gift of rendering the obscure clear; and his thorough grasp of his subject enables him to bring out the real significance of experimental and clinical observations whose connection with the matter in hand was before difficult to appreciate.

The history of the disease is briefly discussed; and then we have, in an interesting and well-arranged chapter, an account of the various experiments and theories with regard to glycosuria, and a discussion of their bearing upon diabetes. The author truly says that there is probably no disease which is so overweighted by theoretical considerations derived from an overwhelming amount of physiological experiment; but, at the same time, by his method of treating these he shows how much there is to be gained from a thorough acquaintance with them as a preliminary to the study of the disease itself. He indicates the gaps which exist in our information and probably prevent us from estimating aright the bearing upon diabetes of some of the experiments from which we are at present able to glean little advantage.

Tables are given of the incidence of diabetes in the various British Colonies. We notice that they do not confirm the opinion of a recent American writer, that there is a greater proportionate number of cases in cold and damp climates.

The section on morbid anatomy consists of the Bradshawe Lecture for 1890. The author is inclined to consider the changes found in the pancreas and semi-lunar ganglia as of great importance, though further observations are at present desirable. To those who are disposed to disparage pathology we may quote his remark that "diabetes, far from having no morbid anatomy, has by later investigations been shown to have one of a very complicated and far-reaching kind." This whole section will repay careful study, and is illustrated by good drawings of the microscopical appearances.

In the chapter on the clinical history, and in the following one on diabetic coma, the chief points are illustrated by short accounts of cases. These cases are well-selected and reported, and add to the value of the text. A fact of some interest is, the liability of diabetics to enteric fever. We would suggest that the directions for testing the urine for sugar, etc., would be better in a short chapter by themselves.

There is a good account of the skin diseases that are found in diabetes. The discussion of diabetic coma which follows occupies considerable space, and is the best account of the subject we have seen. Dr. Saundby thinks that three clinical types of coma must be accepted, though one of them is very rare. He is cautious as to drawing any positive conclusions as to the cause of the coma from the data at present at our disposal, and concludes that "we are unable to determine positively the nature of the toxic substance or the determining causes of the sudden explosion of the fatal terminal symptoms; these phenomena are toxæmic, and in diabetes depend upon the presence in the blood of some substance nearly allied to acetone."

A chapter on diabetes insipidus gives a good account of this hitherto somewhat mysterious complaint. To every chapter is appended a good bibliography.

In conclusion, we may say that those who wish to possess a clear and full idea of the present state of knowledge of diabetes, both from the practical and from the theoretical standpoints, will do well to read this book carefully.

